# A Novel Disulfide-Rich Protein Motif from Avian Eggshell Membranes

**DOI:** 10.1371/journal.pone.0018187

**Published:** 2011-03-30

**Authors:** Vamsi K. Kodali, Shawn A. Gannon, Sivakumar Paramasivam, Sonali Raje, Tatyana Polenova, Colin Thorpe

**Affiliations:** 1 Department of Chemistry and Biochemistry, University of Delaware, Newark, Delaware, United States of America; 2 Department of Chemistry, Towson University, Towson, Maryland, United States of America; University of Crete, Greece

## Abstract

Under the shell of a chicken egg are two opposed proteinaceous disulfide-rich membranes. They are fabricated in the avian oviduct using fibers formed from proteins that are extensively coupled by irreversible lysine-derived crosslinks. The intractability of these eggshell membranes (ESM) has slowed their characterization and their protein composition remains uncertain. In this work, reductive alkylation of ESM followed by proteolytic digestion led to the identification of a cysteine rich ESM protein (abbreviated CREMP) that was similar to spore coat protein SP75 from cellular slime molds. Analysis of the cysteine repeats in partial sequences of CREMP reveals runs of remarkably repetitive patterns. Module **a** contains a **C**-X_4_-**C**-X_5_-**C**-X_8_-**C**-X_6_ pattern (where X represents intervening non-cysteine residues). These inter-cysteine amino acid residues are also strikingly conserved. The evolutionarily-related module **b** has the same cysteine spacing as **a**, but has 11 amino acid residues at its C-terminus. Different stretches of CREMP sequences in chicken genomic DNA fragments show diverse repeat patterns: e.g. all **a** modules; an alternation of **a**-**b** modules; or an **a**-**b**-**b** arrangement. Comparable CREMP proteins are found in contigs of the zebra finch (*Taeniopygia guttata*) and in the oviparous green anole lizard (*Anolis carolinensis*). In all these cases the long runs of highly conserved modular repeats have evidently led to difficulties in the assembly of full length DNA sequences. Hence the number, and the amino acid lengths, of CREMP proteins are currently unknown. A 118 amino acid fragment (representing an **a**-**b**-**a**-**b** pattern) from a chicken oviduct EST library expressed in *Escherichia coli* is a well folded, highly anisotropic, protein with a large chemical shift dispersion in 2D solution NMR spectra. Structure is completely lost on reduction of the 8 disulfide bonds of this protein fragment. Finally, solid state NMR spectra suggest a surprising degree of order in intact ESM fibers.

## Introduction

Disulfide-rich structural proteins (showing half-cystine contents of ∼10% or more) are found in a wide range of biomaterials. For example, keratin-associated proteins in hair and epidermis (containing up to 37% cysteine) form a disulfide-linked matrix around the keratin intermediate filaments [1], [2], [3]. A mitochondria-associated cysteine-rich protein (∼17% cysteine) forms a disulfide-hardened capsule around sperm mitochondria at the flagellar midpiece [4]. From the sea, there are barnacle cements (e.g. cp-20k; ∼18% cysteine) [5], [6], the minicollagens of the stinging organelles of jellyfish [7], and the cysteine-rich proteins that contribute to the slow block to polyspermy in sea urchins [8]. In the aquatic midge *Chironomus*, a series of unusual disulfide-rich silk proteins (∼1750 residues; ∼16% cysteine) are extruded as insoluble fibers [9], [10]. The chorion (eggshell) of the silkmoth *Bombyx mori* contains structural proteins with cysteine contents of up to about 30% [11], [12]. A particularly striking example of a disulfide-rich structural protein is the enormous extracellular DUMPY protein from the *Drosophila* extracellular matrix with >25,000 amino acids, almost 500 domains, and >1200 disulfide bonds [13], [14].

**Figure 1:**
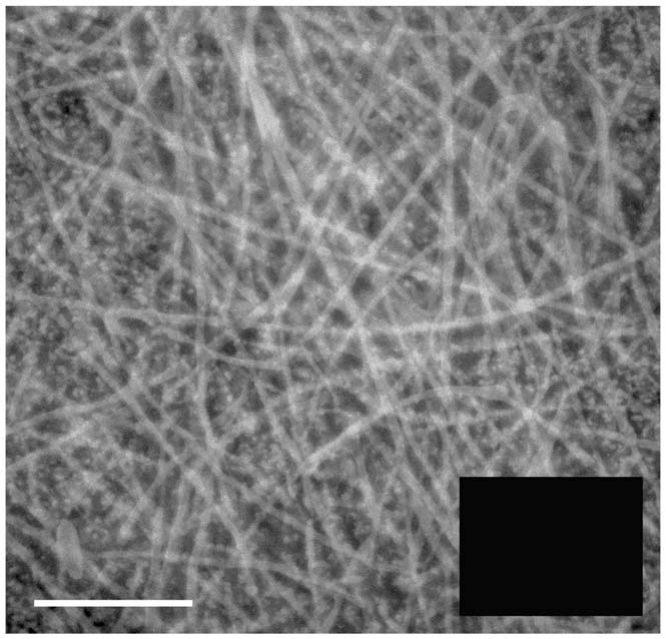
Fluorescence microscopy of chicken ESMs. The main panel shows reduced ESM labeled with monobromobimane (see Methods; the bar is 10 microns long). Membranes that were not pre-reduced with DTT do not show significant fluorescence when treated with monobromobimane and examined under the same magnification and instrument settings (dark inset).

This work describes a disulfide-rich biomaterial that is both commonplace and cryptic. Under the shell of a chicken egg is a parchment-like membrane formed from two similar layers of interwoven proteinaceous fibers [15]. These cysteine-rich (∼10% cysteine, Table S1 and Figure 1) fibers are in some way layered over egg white in the avian oviduct prior to the deposition of the mineralized shell. While the ultrastructure of these eggshell membranes (ESM) has been known for decades [16], [17], [18], key aspects of their nature and fabrication remain unknown. Critically, there is no consensus as to their protein composition: they have been described as containing (ovo)keratins, elastins, and collagens, although evidence against major involvement of each of them has been adduced [15], [17], [19], [20], [21]. This confusion is readily understandable because the membranes are extensively, and irreversibly, crosslinked with the lysine-derived desmosine and isodesmosine linkages. Hence the fibers are intractable to reducing agents and resist analysis by the standard methods of the protein chemist [15], [21], [22], [23].

Our interest in ESMs came from the discovery of a facile catalyst for protein disulfide bond generation that was found both in chicken oviduct tissues and in avian egg white [24], [25]. We found that this Quiescin-sulfhydryl oxidase (QSOX) was an efficient oxidant of reduced peptides derived from ESM. Here, we show that many of these cysteine-containing peptides derive from a novel disulfide-rich structural protein. This **c**yst(e)ine-**r**ich **e**ggshell **m**embrane **p**rotein (CREMP) shows a modular sequence architecture that is formed from multiple disulfide-containing units presenting a remarkably conserved set of intervening residues. This extreme conservation complicates assembly of full-length sequences from genomic DNA, and has undoubtedly slowed recognition of this new structural motif. A cDNA from the avian genome has been annotated as similar to spore coat proteins of *Dictyostelium discoideum* and *Polysphondilium pallidum*, but these latter proteins have sequence repeats [26] that are much more variable than the CREMP protein from birds and lizards described here. NMR experiments performed on recombinant avian CREMP modules and on the ESM fibers themselves indicate that both show striking local structural order. These studies lay the groundwork for an eventual understanding of the role of the CREMP protein(s) in eggshell membrane: a biomaterial critical to the assembly and biological fitness of eggs of birds and reptiles.

## Materials and Methods

### Materials

Chicken eggs were purchased from a local grocery store. The eggs were cracked and the insides of the shells washed thoroughly with a stream of distilled water. The fused inner and outer eggshell membranes were then manually pulled away from the calcified shell and the pieces stored under water at 4°C until use. Avian QSOX was isolated from egg white as described previously [24].

### Microscopy

Chicken ESMs were washed extensively with distilled water and reduced using 10 mM dithiothreitol (DTT) in 50 mM phosphate buffer, 1 mM EDTA pH 7.5 for 30 min at room temperature. After reduction, excess reagents were removed using distilled water and the membranes were incubated with thiol-specific fluorescent dyes. Initial experiments using fluorescein-5-maleimide and tetramethylrhodamine-6-maleimide showed non-specific binding of the dyes to both untreated and reduced ESMs. For the experiments described here, ESMs were incubated with 10 µM monobromobimane in phosphate buffer for 5 minutes at room temperature. Excess dye was removed using distilled water and the membranes were mounted with Vectashield (Vector laboratories) on a glass slide under an 18 mm glass coverslip. Images of reduced and oxidized membranes were obtained on a Leica DMRXA2 epifluorescence microscope (Leica Microsystems) fitted with a Hamamatsu ORCA-ER camera (Hamamatsu). Images were initially acquired using the Openlab 5.5.0 software (Improvision) and processed using Photoshop (Adobe).

### Reduced ESM peptic peptides

Membranes were washed extensively with water, homogenized in water with a small blender and lyophilized. The dry pink powder (0.5 g) was mixed end over end at room temperature with 50 mL of 200 mM potassium phosphate buffer containing, 25 mM DTT, and 0.3 mM EDTA adjusted to pH 7.5. The membrane solids were recovered by filtration on a Buchner funnel lined with a Whatman No. 1 filter paper, and then washed with 5×5 mL aliquots of 0.1% v/v acetic acid to remove excess DTT. The membrane fragments were then lyophilized and stored at −20°C. ESM peptic peptides to be tested as substrates of avian QSOX were prepared by treating the reduced powder (100 mg, as above) with 2 mg of pepsin in 10 mL of stirred 5% formic acid at 37°C. After 3h, the solution was neutralized with concentrated KOH before being used as a substrate in the oxygen electrode. Throughout this work thiol titers were determined using 5,5′-dithiobis(2-nitrobenzoate) (DTNB) as described earlier [24], [27].

### Solid-state NMR spectroscopy

Membrane pieces (either untreated or reduced with DTT) were packed directly into a solid-state 4.0 mm NMR rotor and sealed using end caps and driving tips. The samples were spun under boil-off nitrogen gas maintaining the sample anaerobic and controlling the temperature inside the NMR probe. Magic angle spinning (MAS) solid-state NMR spectra were acquired at 14.1 T (600 MHz) and 26°C on a narrow bore Varian InfinityPlus instrument outfitted with a 4.0 mm triple resonance T3 probe; Larmor frequencies are 599.54 MHz for ^1^H, and 150.77 MHz for ^13^C. All spectra were collected at the MAS frequency of 10.000±0.001 kHz using a Varian MAS controller. The temperature was calibrated for this probe at different MAS frequencies using a lead nitrate temperature sensor [28] and the sample was maintained to within ±0.5°C throughout the experiments using the Varian temperature controller. ^13^C chemical shifts were referenced to the downfield peak of adamantane (40.3 ppm with respect to 4,4-dimethyl-4-silapentane-1-sulfonic acid; [29]). The ramped-CP sequence was used with 1.25 ms contact time; the ^1^H radio frequency field strength was 50 kHz, the ^13^C field was linearly ramped 80–100% with the center of the ramp being 40 kHz. The ^1^H 90° pulse width was 3.1 µs. The ^1^H decoupling was performed using a two-pulse phase-modulated decoupling sequence [30] with a 80.6 kHz ^1^H rf field strength. The recycle delay was 5 s. The spectra were processed in Spinsight by Fourier transform and phase correction; no apodization was applied.

### Isolation of ESM peptides

Approximately 22 mg (wet weight) of ESM pieces diced into approximately 1 mm squares were incubated at 37°C for 45 min in 3 mL of 500 mM ammonium bicarbonate buffer (pH 8.6) containing 6 M guanidine HCl. The membranes were subsequently reduced using 6 mM DTT. After 4 h, free thiols were alkylated in the dark for 30 min using 500 mM sodium iodoacetate. The membranes were rinsed three times with 50 mM ammonium bicarbonate buffer, pH 7.6 and then resuspended in 2 mL of 6 M guanidine HCl at 37°C for 1 hr. The suspension was diluted with 4 mL of 50 mM ammonium bicarbonate, pH 7.6, followed by the addition of 0.4 mL of a 2 mg/ml solution of trypsin. The sample was largely solubilized after constant shaking for 23 h at 37°C. The mixture was applied to a 30 mg Strata-X SPE column (Phenomenex, Torrance, CA) and the column washed with 1 mL of water. Peptides were eluted with 1 mL of a mixture of acetonitrile/methanol/formic acid (1∶1∶0.1 v/v/v) and dried by vacuum centrifugation. Peptides were then redissolved in 0.1% formic acid and analyzed by HPLC-MS using a 1×100 mm Waters Acquity UPLC BEH C18 column at 40°C with either a Waters QTOF2 quadrupole time-of-flight mass spectrometer using data dependent scanning or a Waters OrbiTrap Velos. HPLC analysis was performed using a 95∶5 mixture of solvents A (0.1% formic acid) and B (0.08% formic acid in methanol), respectively. After an initial hold of two minutes with 95% solvent A (0.1% formic acid in water) and 5% solvent B (0.08% formic acid in methanol), the chromatogram was developed with a linear gradient up to 60% solvent B at 42 min. The resulting MS data file was processed using Waters ProteinLynx software to generate PKL files which were then submitted to the Open Mass Spectrometry Search Algorithm (OMSSA; [31]). Peptide sequences with the least E-value for each spectrum were filtered and used to perform BLAST searches against the chicken RefSeq database.

### CREMP constructs

**Figure 2:**
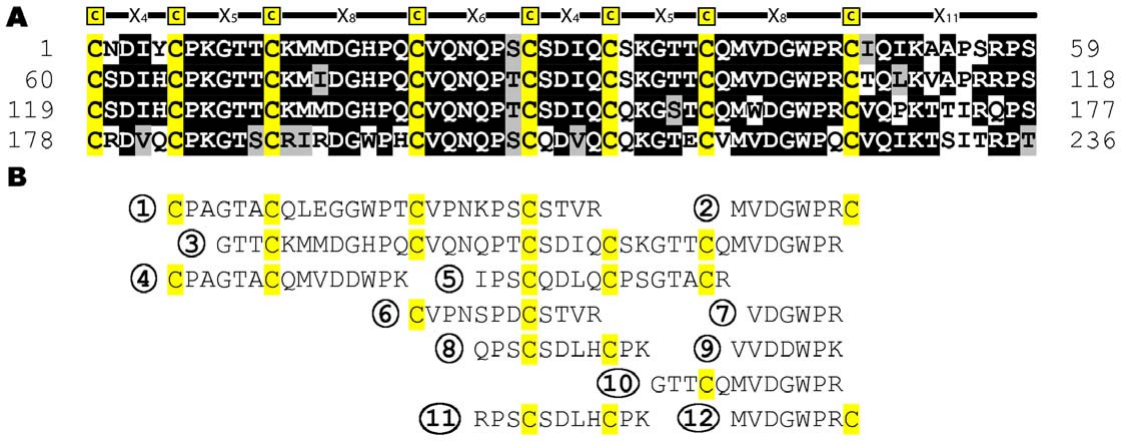
Protein and peptide sequences of *Gallus gallus* CREMP. Panel A shows the protein sequence of *Gallus gallus* CREMP obtained upon translating the EST sequence. Each line constitutes a single CREMP repeat, and the four repeats found in the EST are aligned to each other using ClustalW. Cysteine residues are highlighted in yellow. Panel B shows 12 CREMP peptides found in this study. Each peptide is numbered and aligned with the EST sequence.

An EST encoding four complete CREMP repeats (UD Chick EST database Accession Number: pgr1n.pk001.g1) was a kind gift from Dr. Joan Burnside, Delaware Biotechnology Institute, University of Delaware. The sequences of primers used in this study are listed in Table S2. The first two complete repeats of this EST sequence (the first two lines of Figure 2A) were subcloned between *Bam*HI and *Eco*RI sites of the pTrc His A vector (Invitrogen) to generate a CREMP-2Rep-pTrc construct. This construct was further modified (to give CREMP-2Rep-TEV-pTrc) by the inclusion of a TEV protease site (ENLYFQS) immediately upstream of the CREMP repeats with an intervening GG dipeptide to assure efficient cleavage. All constructs were tested by restriction enzyme digestion for the presence of the insert. In-frame ligations, and the absence of unwanted mutations, were confirmed by automated DNA sequencing.

### Expression of CREMP-2Rep

Initial attempts to express and purify the protein from *Escherichia coli* BL21 Star (DE3) cells (Invitrogen) resulted in insoluble aggregates, presumably because this strain does not support the cytosolic formation of disulfide bonds. CREMP-2Rep protein was routinely expressed and purified from *E. coli* Rosetta-gami (DE3) cells (Novagen). Briefly, bacterial cultures were inoculated with overnight starter cultures and grown at 37°C until they reached an OD_600_ of ∼0.8 before induction with 1 mM IPTG for 6 h at 37°C. For the expression of isotope-labeled protein used for the NMR experiments, our attempts at growing *E. coli* Rosetta-gami (DE3) cells in ^15^NH_4_Cl-enriched minimal media were unsuccessful. However, a similar *gor/trx* mutant co-expressing *E. coli* DsbC in the cytoplasm (*E. coli* Shuffle T7 Express; New England Biolabs Inc.) was employed in conjunction with the protocol described earlier [32]. Cultures were grown at 37°C until they reached an OD_600_ of ∼0.8 and centrifuged at 5000×*g* for 15 min at 4°C. The pellet was washed with phosphate buffer (pH 7.5) and resuspended in 0.25 volumes of isotopically labeled minimal media. The culture was allowed to grow for an hour in minimal media followed by induction with 1 mM IPTG for 6 h at 37°C.

### Purification of CREMP-2Rep

Cell pellets were resuspended at 0.5 g wet weight/mL of lysis buffer (50 mM potassium phosphate, pH 7.5, containing 300 mM sodium chloride, 1 mM phenylmethylsulfonyl fluoride and 10 µM leupeptin). Cells were lysed by passing the suspension twice through a French pressure cell at 10,000 psi followed by three 30 s periods of sonication on ice. The resulting lysate was clarified by centrifugation (17,000×*g* for 30 min at 4°C) and incubated with Ni-NTA resin (Invitrogen) at 4°C for 1 h. The suspension of resin and crude extract was then poured into an empty column and unbound proteins were allowed to flow-through. The column was then washed with four column volumes of lysis buffer followed by two additional washes with lysis buffer containing 10 mM imidazole at pH 6.0. Bound proteins were eluted in 5 mL fractions using 20 mL each of 100, 200, 300 and 500 mM solutions of imidazole in phosphate buffer at pH 6.0. During purification, a flavoprotein contaminant (determined to be *E. coli* alkylhydroperoxide reductase by mass spectrometry analysis; data not shown) consistently coeluted with CREMP-2Rep. Contamination was minimized by introducing an additional washing step using 50 mM imidazole before elution. A subsequent size exclusion chromatography (using a 1×30 cm Superdex 200 column) removed this contamination entirely. Fractions containing CREMP-2Rep protein were pooled and concentrated using Amicon centrifugal filter devices (Millipore; MWCO of 10 kDa). The thiol titer of purified recombinant CREMP-2Rep, determined using 0.5 mM DTNB under native or 1% SDS denaturing conditions, showed that all cysteines were oxidized.

CREMP-2Rep protein that incorporated a TEV protease site was treated as follows: CREMP-containing fractions from the Ni-NTA column were pooled and dialyzed against lysis buffer to remove imidazole. The protein was incubated at 4°C overnight with 10% by weight of His-tagged TEV protease [33], [34]. The reaction mixture was incubated with Ni-NTA resin for 0.5 h at 4°C and poured into an empty column. A small amount of TEV-digested CREMP-2Rep was collected in the flow-through. A solution of 50 mM imidazole in 50 mM potassium phosphate buffer at pH 6.0 was used to extract the remaining amount of CREMP-2Rep from the column leaving the TEV protease firmly retained. The purified protein was dialyzed against phosphate buffer to remove imidazole and concentrated using Amicon centrifugal devices as above. Routinely, the protein was stored at 4°C in phosphate buffer, pH 7.5, containing 1 mM EDTA.

### Solution NMR spectroscopy

Solution heteronuclear single quantum coherence (HSQC) NMR spectra of CREMP were acquired at 25°C at 14.1 T (600 MHz) on a Bruker Avance spectrometer outfitted with a triple-resonance inverse detection Cryoprobe; Larmor frequencies were 600.13 MHz for ^1^H, 150.91 MHz for ^13^C, and 60.82 MHz for ^15^N. Chemical shifts were referenced to the residual water resonance at 4.7 ppm. The 2D spectra were acquired as (1024×64) complex matrices; States-TPPI [35] was used for frequency discrimination in the indirect dimension. Thirty two FID's were added per each t1 increment. The spectra were processed in NMRPipe [36] by sine bell square apodization function shifted by 60^°^, Fourier transformation, and phase correction. The spectra were linear-predicted to twice the number of experimental points in the ^15^N dimension. Spectral analysis was performed in Sparky [37].

## Results and Discussion

### Chicken ESMs are comprised of disulfide-rich protein fibers

Figure 1 shows a fluorescence micrograph of chicken ESM. The membranes were reduced with DTT and then stained with the thiol-specific reagent monobromobimane (see Methods). Without pre-reduction membranes exposed to this alkylating agent do not show the strong blue fluorescence of the bimane-thiol adduct (see inset to Figure 1). ESM fibers are known to emerge from tubular gland cells in the isthmus region of the oviduct and then wound around the surface of a rotating egg white prior to the deposition of the calciferous shell [15], [18]. The fibers are deposited sequentially in inner and outer layers: both are similar in terms of general morphology and amino acid composition [15] and are treated as one for the work described here. ESMs stripped from washed broken eggs contain undetectable levels of free thiols using DTNB (data not shown).

### Chicken ESMs contain a novel cysteine-rich protein

The extensive crosslinking of ESMs with desmosine and isodesmosine complicates the effective characterization of the membranes and precludes the separation and analysis of its component proteins. Partial digestion of reduced alkylated membranes with trypsin followed by MS/MS analysis indicated the presence of several globular proteins already found to be associated with ESMs (e.g. ovocalyxin, lysozyme and lysyl oxidase; Table S3, [38]). In terms of structural proteins, our small-scale peptide analysis suggested the presence of a number of collagens (Table S3). Indeed, the presence of collagen has been inferred from prior immunohistochemical staining [39], [40], [41]. However, a recent report suggesting that collagens make up ∼90% of ESM dry weight [42] is not supported by earlier determinations of the amino acid composition of ESM (Table S1). For example, ESMs contain only 9.5±2.8% glycine, whereas the canonical G-X-Y repeat of collagen triple helical regions contribute to an average glycine content of avian collagens (I, V and X) of ∼25%. Further, the high cysteine content of ESM (10.1±0.7%) is clearly incompatible with the average of 0.9% cysteine for these avian collagens. Elastin is similarly excluded from being a major constituent of ESM because of its particularly low cysteine content (∼0.3%).

While avian extracellular keratins average 7.5% cysteine, we only found one keratin-derived peptides by MS/MS consistent with previous arguments [18] that keratins are not major components of ESM. What, then, is the origin of the high cysteine content of ESM? We found a number of cysteine-containing peptides from ESM digests that resembled hypothetical protein fragments annotated as being “similar to spore coat protein SP75”. Related sequences were found in three chicken contigs (Figure S1 A–C) and in an EST from a chicken oviduct library (Figure 2A). Figure 2B shows 12 peptides that could be placed, often multiple times (when minor differences in the sequence are allowed), in this stretch of 236 amino acid residues. This previously unrecognized and unusual cysteine-rich protein from chicken egg shell membranes is abbreviated CREMP. Parenthetically, a 26-residue peptide from Japanese quail oviducts isolated by Li et al. is clearly derived from a CREMP protein.

### CREMP repeats and modules

Based on the sequences present in the genomic and EST databases, it appears that some portion of CREMP is comprised of a series of remarkably conserved repeating units (arranged as four consecutive lines in Figure 2A). Here, the repeating unit is:

**C**-X_4_-**C**-X_5_-**C**-X_8_-**C**-X_6_-**C**-X_4_-**C**-X_5_-**C**-X_8_-**C**-X_11_

where X represents any amino acid, except cysteine, and the subscript represents the number of intervening residues. Not only is this pattern repeated multiple times within each sequence fragment, but there is also a surprising conservation of non-cysteine residues between consecutive repeats (Figure 2A). This high degree of repetition and inter-repeat similarity has undoubtedly hindered the assembly of full-length sequences from genomic DNA. Indeed, these regions of the genome remain incomplete in both chicken and zebra finch (*Taeniopygia guttata*) genomes (see later). For these reasons we do not know how many repeats are contained in this ESM protein. CREMP might be present in multiple isoforms in the chicken genome (e.g. analogous to the multiplicity of keratin-associated proteins in vertebrates [3]). CREMP proteins might also be thousands of residues long, and hence the various EST and contig sequences might represent fragments of a single protein. Here, our focus is on the characterization of a protein fragment, rather than attempts to secure the sequence of a full length protein.

One observation argues that the type of sequence shown in Figure 2A is likely to be a major component of avian ESM. The amino acid composition of the intact eggshell membranes bears a statistically significant similarity to that of the average composition of all available CREMP repeats (deduced from EST and contig sequences; Figure S2A and B). The average amino acid composition of chicken ESMs is closer to the composition of CREMP than collagens, keratins and elastin.

### Solid-state NMR of eggshell membranes demonstrates molecular order

**Figure 3:**
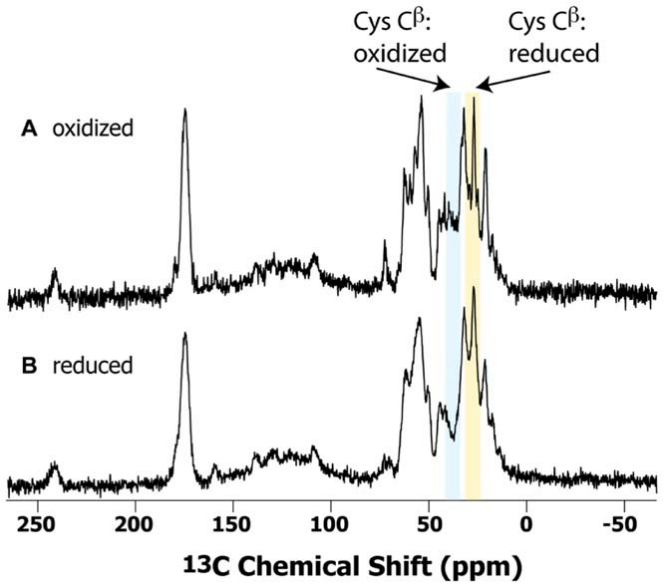
^13^C solid-state CPMAS NMR spectrum of chicken ESM. a) oxidized sample (4096 scans); b) reduced sample (8192 scans). The resolved peaks display narrow lines, ∼0.5 ppm, indicating a high degree of order. Upon reduction, the spectral intensity in the region of 42±5 ppm (C^β^ chemical shifts of oxidized Cys; blue bar) is markedly decreased accompanied by the corresponding increase in the spectral intensity around 27±5 ppm (C^β^ chemical shifts of reduced Cys; yellow bar).

A striking observation is that the intact ESM appears to exhibit marked structural order when observed by ^13^C natural abundance solid-state NMR. The cross polarization magic angle spinning (CPMAS) spectra of the naturally occurring membranes presented in Figure 3A exhibit narrow lines, of the order of 0.5 ppm for the resolved peaks. Thus these membranes are not random assemblages of polypeptide chains crosslinked by multiple disulfides, but are significantly structured. Upon reduction the spectral lines broaden (Figure 3B) and the fine structure of the peaks in the aliphatic region disappears, suggesting that the inherent structural order is perturbed. Furthermore, upon reduction of membranes by DTT, the spectral intensity in the region of 42±5 ppm (C^β^ chemical shifts of oxidized Cys) is markedly decreased and is accompanied by a corresponding increase in the spectral intensity around 27±5 ppm (C^β^ chemical shifts of reduced Cys). Interestingly, the original spectrum of the oxidized form is not restored even after 12 hours of data collection suggesting that the membranes remain reduced over this time interval. Future work will address the intrinsic structure of the membranes by solid state NMR spectroscopy. However, these studies are beyond the scope of the current work; it is currently not feasible to introduce isotopic labels into native ESM fibers of a laying hen.

### Expression and Purification of CREMP

These findings encouraged us to pursue the expression of a fragment of CREMP sequence. Since the repeats exhibit a high degree of similarity, we arbitrarily chose the first two complete repeats in Figure 2A (residues 1–118; termed CREMP-2Rep hereafter) for our studies. The sequence (Figure S3) was first subcloned into an expression vector pTrc His A for expression in *E. coli*. Expression trials of this 8-disulfide protein fragment in *E. coli* BL21 Star (DE3) cells were unsuccessful with the formation of insoluble aggregates. Hence we used a *gor/trx* mutant strain (*E. coli* Rosetta-gami (DE3)) that allows cytosolic disulfide bond generation. Protein purification was performed using Ni-NTA affinity chromatography utilizing the N-terminal hexahistidine tag followed by size exclusion chromatography (see Methods). To facilitate the removal of the N-terminal tag, a TEV protease site was introduced immediately upstream of the CREMP-2Rep sequence by site-directed mutagenesis (Figure S3). However, TEV protease failed to cleave in the absence of a reductant (data not shown). This behavior is likely due to steric restrictions conferred by the presence of a disulfide bridge adjacent to the TEV protease recognition sequence. Introduction of a glycine dipeptide spacer upstream of the first cysteine of the CREMP-2Rep resulted in efficient cleavage.

**Figure 4:**
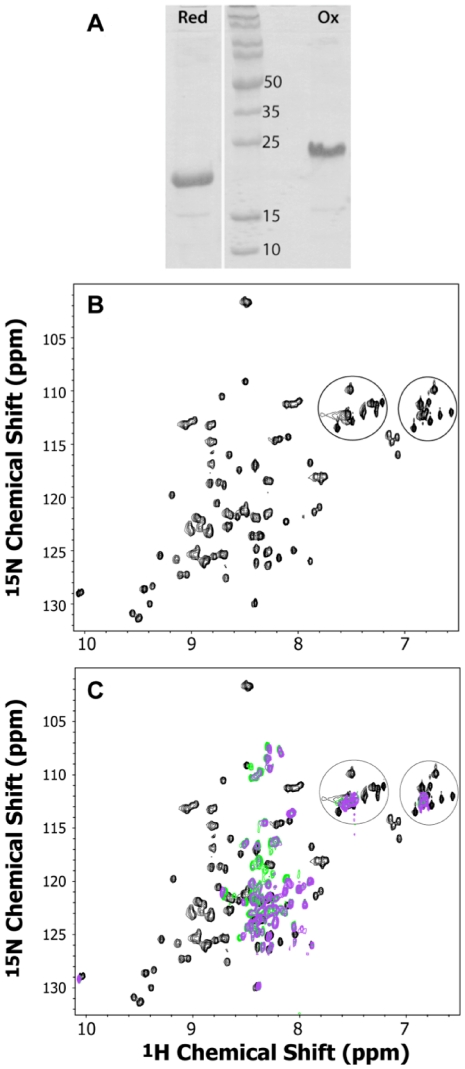
Biophysical characterization of CREMP-2Rep. Panel A: SDS-PAGE gels of reduced and oxidized forms of CREMP. Panel B: 2D ^1^H-^15^N HSQC spectra of recombinant CREMP2 protein as isolated. Panel C: Overlay of three HSQC spectra (black, oxidized sample; green, after 2 mM THP addition; purple, after 4 mM THP addition). The cross peaks in circles are mostly side-chain correlations folded into the backbone amide region of the spectrum.

Purified CREMP-2Rep is a tractable, soluble, protein in phosphate buffer pH 7.5 containing 1 mM EDTA. The protein, as isolated, contained no free thiols (see Methods) and a full complement of 8 disulfide bonds. SDS-PAGE under non-reducing conditions gave a single dominant band (Figure 4A). However the behavior of CREMP-2Rep on SDS PAGE was distinctly anomalous. Normally, the presence of disulfides constrains the conformational excursions of the polypeptide chain of a typical globular protein in the presence of SDS. A pure sample of an oxidized protein is thus expected to migrate faster than its reduced counterpart. In contrast to these expectations, reduced CREMP-2Rep runs much faster than the oxidized protein (with an apparent MW of 17 kDa compared to 23 kDa; Figure 4A). These data are consistent with CREMP-2Rep being significantly anisotropic in solution.

### Biophysical characterization of recombinant protein

Further strong evidence that CREMP-2Rep has a defined protein fold is provided by 2D ^1^H-^15^N HSQC NMR of CREMP-2Rep from which the N-terminal His-tag had been removed by TEV protease digestion (see Methods). Figure 4B shows a well resolved series of sharp resonances, which are well dispersed and consistent with a highly ordered structure. At least 113 resonances out of a total of 121 expected were observed. This is both notable and unexpected because the two repeats show 86% sequence identity. Recombinant CREMP-2Rep is thus well ordered: it is not a random mixture of disulfide-bonded isomers. Treatment of the protein with the disulfide reductant tris(hydroxypropyl)phosphine (THP; [43]) leads to a collapse of the widely dispersed residues and reversion to the classic pattern found in disordered molten globular proteins (e.g. see the purple resonances in Figure 4C). Disulfide reduction is also accompanied by an approximate 2-fold decrease in the protein fluorescence when excited at 280 nm (Figure S4). Each repeat in CREMP-2Rep contains an equivalently placed tryptophan residue (at positions 45 and 104 in Figure 2A). The high quality of the HSQC spectra of the oxidized CREMP-2Rep protein indicates that full resonance assignments and structure determination will be feasible from a series of 3D heteronuclear correlation experiments; these studies are currently ongoing in our laboratory.

### Reduced ESM peptides and CREMP-2Rep are substrate of chicken QSOX

An aggregate of reduced peptic peptides from chicken ESM (300 µM thiols; see Methods) proved to be an efficient thiol substrate of the avian sulfhydryl oxidase QSOX1 that is found both in egg white and in avian oviduct tissue [24]. The apparent turnover at 300 µM thiols was found to be 1050±70 thiols oxidized/min (not shown). At a comparable thiol concentration reduced CREMP-2Rep shows an initial turnover number of 860 thiols/min (Figure S5). Under these conditions essentially all of CREMP-2Rep thiols are oxidized after 20 min. Hence reduced CREMP-2Rep is a viable substrate of QSOX1.

### Non-metazoan CREMP homologs

**Figure 5:**
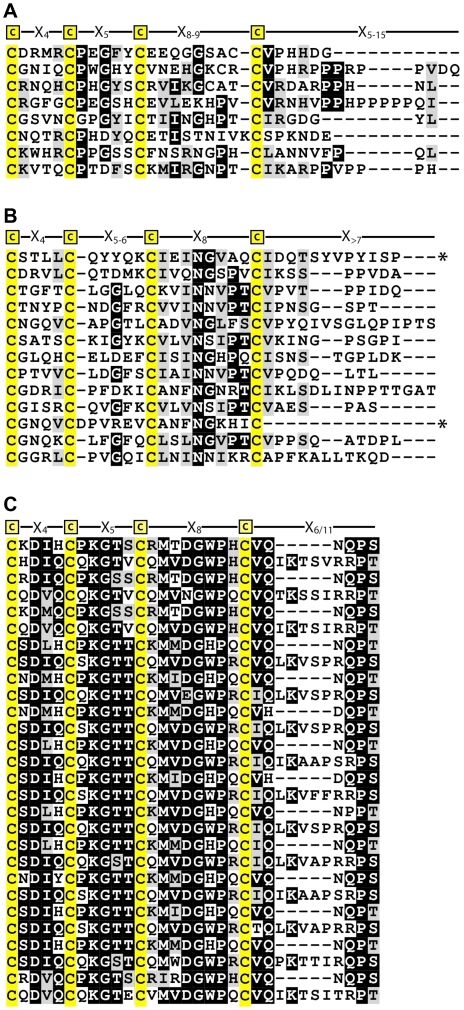
Comparison of spore coat proteins from slime molds to chicken CREMP. Panels A and B show ClustalW alignments of the C4C modules of spore coat protein SP75 from *Dictyostelium discoideum* and *Polysphondylium pallidum*, respectively. For comparison, panel C shows the alignment of the modules of *Gallus gallus* CREMP. Cysteine residues that are absolutely conserved in each module are highlighted for clarity. The cysteine-based pattern exemplified by the modules is depicted at the top of each alignment.

The CREMP sequence is currently annotated as similar to spore coat proteins (SP75) from the slime molds *Polysphondylium pallidum* and *Dictyostelium discoideum*. There are several of these cysteine-rich (7–13% C) proteins that are believed to form intra- and inter-chain disulfide bonds as they integrate with an intervening cellulosic layer around the spore cell surface [44]. West and coworkers recognized that these proteins contain mucin-like repeats, basic proline repeats, and six types of cysteine motifs/spacings [26], [44], [45]. One of these latter motifs has a spacing of four cysteines summarized by **C**-X_4_-**C**-X_5_-**C**-X_6-10_-**C** [26], [44]. These repeats in SP75 proteins from *P. pallidum* and *D. discoideum* are shown in Figure 5, together with all of the recognized repeats from chicken databases. While there is clearly a strong conservation of cysteine spacing, the slime mold repeats are much more variable in terms of their C-termini and their intervening residues (compare panels A and B with panel C).

### Insight into the evolution and structure of CREMP proteins

The protein sequence in Figure 2A shows a string of **C**-X_4_-**C**-X_5_-**C**-X_8_-**C**-X_6_-**C**-X_4_-**C**-X_5_-**C**-X_8_-**C**-X_11_ repeats that reinforced the idea that this 59- residue pattern was the fundamental unit of structure. However, this prototypical repeat is actually comprised of two related halves that we will call **modules**. The N-terminal **a** module is almost invariably **C**-X_4_-**C**-X_5_-**C**-X_8_-**C**-X_6_; the C-terminal **b** module is here **C**-X_4_-**C**-X_5_-**C**-X_8_-**C**-X_11_. Although the first sequence we found (Figure 2A) features a strict **a-b** alternation of modules, other sequences show patterns like (**a-b-b**)_n_ or (**a**)_n_ (Figure S1, panels A and C respectively).

**Figure 6:**
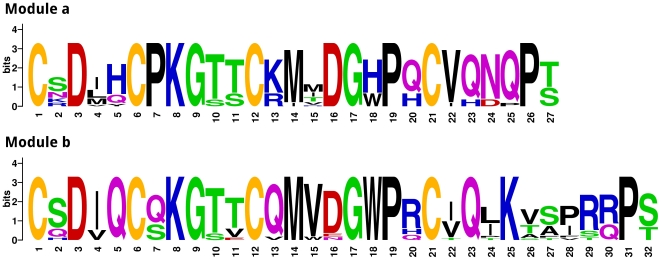
Conservation of amino acid residues in a and b modules of chicken CREMP. All available chicken CREMP repeats in the (a-b)_n_ format were extracted and the conservation of amino acid residues in each module is plotted using WebLogo.

Figure 6 shows the conservation of amino acid residues in all of the paired **a** and **b** modules of chicken CREMP (including the **a-b** repeats shown in Figure 2A and Figure S1). There are notable identities between modules, including the spacing of cysteines, and the presence of the absolutely conserved residues D3, K8, G9, G17 and P19. However, there are also distinct differences between **a** and **b** modules. An obvious difference is the length of the two modules (Figure 6). Further, a P7 and P26 are always found in the **a** module, but never in the **b** module. In addition, a conserved lysine is found at position 25 only in the **b** module. To explore a possible evolutionary relationship between **a** and **b** modules we made them of equal length by arbitrarily trimming 5 residues from the C-terminus of the **b** module. When the resulting sequences were probed via ClustalW, the **a** and **b** motifs were rigorously segregated in a phylogenetic tree shown in Figure S6. A corresponding analysis of untrimmed sequences again gave complete segregation (data not shown). Hence the 59 residue repeat likely derives from gene duplication of a primordial module sequence. Subsequent duplications could then generate a variety of module patterns.

### Zebra finch

**Figure 7:**
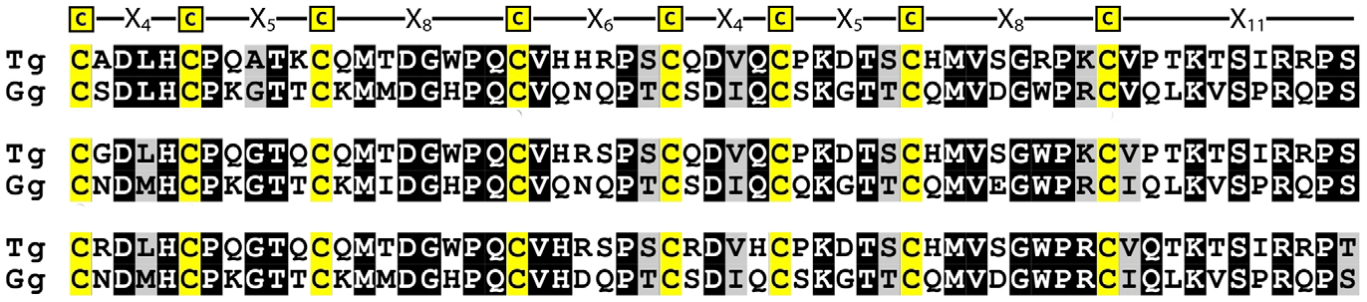
Alignment of chicken and finch CREMP repeats. Three contiguous CREMP repeats from chicken (Gg) and finch (Tg) contigs were extracted and aligned using ClustalW. Each block of alignment represents a single repeat. Cysteine residues are highlighted for clarity and the cysteine-based pattern is shown on the top.

The only other bird for which a substantially complete genome sequence is available is the zebra finch (*Taeniopygia guttata*). As found with the chicken genome, only partial finch CREMP sequences are available (Figure S7). Figure 7 shows an alignment of 3 consecutive **a**-**b** repeats from both finch and chicken CREMP. The bird sequences share 56% identity and show the same defining differences in **a** and **b** modules. As observed with the chicken sequences, the **a** and **b** modules of finch CREMP again segregate on ClustalW analysis.

### Green Anole Lizard

**Figure 8:**
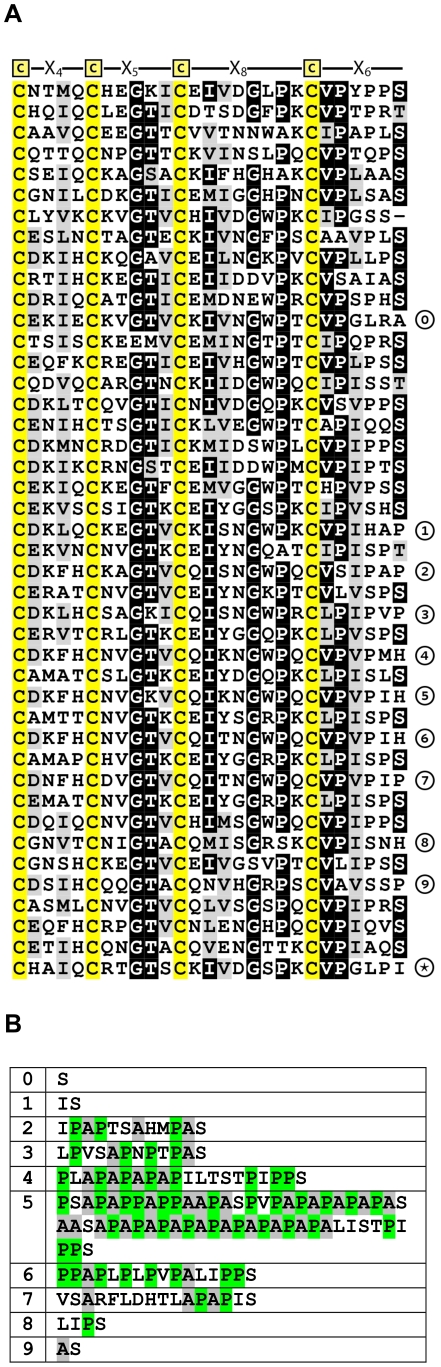
Repeats of Lizard CREMP. Sequences of CREMP modules derived from the whole genome shotgun sequence (NCBI Accession AAWZ02022960) of *Anolis carolinensis* are aligned using ClustalW. The sequence is comprised of predominantly 27-residue modules ending with an X_6_ spacing. At the numbered positions additional residues are present: these inserts (labeled 0–9) are depicted in panel B. The asterisk at the bottom right denotes the end of the CREMP module sequences. Cysteine residues in panel A are highlighted in yellow, and the alanine and proline residues in panel B are highlighted in grey and green.

Eggs from the lizard, *Anolis carolinensis*, contain fibers woven into an ESM comparable to the avian cases [46] and logically would contain at least some of the proteins found in avian ESM. The sequence fragment derived from genomic DNA is clearly a CREMP with a striking repetition of modules (Figure 8). The first 21 modules are all basically of the **a** type (with two minor deviations: a deletion of one amino acid in the 7^th^ module and an extra amino acid at the end of the 12^th^ module. Curiously, even-numbered modules 22–28 progressively lengthen at their C-terminus (reaching an insertion of 59 residues for module 28; Figure 8A). These insertions are strikingly proline- and alanine-rich including runs of AP repeats Figure 8B. This feature then disappears by module 38 (Figure 8B).

### Conclusions

The ESM serves as a critical component of calcified eggs of birds. It provides an additional barrier to microbial egress, furnishes the scaffold on which the calcified shell is deposited and likely provides the foundation for the osmotic barrier of the egg [15]. The fibrous construction of the two layers of the ESM has been recognized for decades and similar structures have been found in egg-laying lizards and in the fossilized eggs of theropod dinosaurs [19], [47]. In spite of its importance, comparatively little is known about the composition and fabrication of these fascinating fibrous biomaterials.

A major component of chicken ESM appears to be fabricated from protein modules containing 4 cysteine residues exhibiting a marked similarity in their intervening amino acid residues. Initially the pattern was recognized with a stretch of CREMP sequence that showed a strict alternation of **a-** and **b-** type modules. However, examination of additional sequences shows that this alternation is not a requirement. While **a-** and **b-** type modules are clearly related, they segregate completely in a ClustalW analysis. We have also identified CREMP sequences in the genomes of Zebra finch and in an oviparous lizard; they are likely widely represented in egg laying birds and reptiles. We have yet to find evidence of these sequences in monotreme genomes.

Somewhat surprisingly, four consecutive modules of chicken CREMP can be readily expressed in *E. coli* and form a highly anisotropic protein containing the expected complement of 8 disulfide bridges. Reduction yields a more globular structure. Further definitive evidence for a well-structured protein fragment is the large chemical shift dispersion in 2D NMR experiments for almost all of the expected resonances of a 118 residue piece of oxidized CREMP. Although one might anticipate that the arrangement of proteins within the ESM of the chicken would lead to very broad lines in solid state NMR, the fibers appear strikingly ordered by this technique.

Clearly, obtaining full length CREMP sequences will represent a major step in our understanding of the construction of ESM fibers. A more immediate goal is the determination of the 3D structure and disulfide connectivity of an **a-b-a-b** segment. Efforts to crystallize this 4-module fragment have proved unsuccessful, and so we are undertaking a structural determination by NMR following the insights gained by earlier workers studying epidermal growth factor and EGF-like domains (e.g. [48], [49], [50]. Finally, we hope that the work described in this paper will renew interest in the nature and fabrication of these intriguing ancient modular biomaterials.

## Supporting Information

Figure S1 **CREMP sequences derived from different** ***Gallus gallus*** **contigs.** The sequences shown in A, B and C are derived from the contigs NW_001473877, NW_001479668 and NW_1475627 respectively. The dashed lines separating sequences in A and B represent a stretch of unassigned nucleotides in the contig. The sequence shown in the second part of A is deposited in the RefSeq database under the accession number XP_001236415.
(TIFF)

Figure S2 **Comparison of the amino acid composition between chicken ESM and CREMP repeats and of CREMP to other structural proteins. Panel A:** For chicken ESMs, an average of the literature values for amino acid composition in Table S1 was used. For CREMP, all available repeats were summed using the ProtParam tool (http://expasy.org/tools/protparam.html) to calculate the amino acid composition. To compare data obtained by acid hydrolysis with compositions deduced from gene sequences the content of ASP and ASN and GLU and GLN are aggregated. Where available, the amounts of Hyl and Hyp were added to Lys and Pro, respectively. Trp was not included because amino acid analyses for this amino acid were unavailable. **Panel B:** Euclidian distances between average amino acid composition of ESMs and other proteins were calculated as described previously [51]. The values for chicken ESM and for CREMP were as in panel A. For keratin, an average amino acid composition of chicken feather keratins 1, 3 and 4 (RefSeq Accession Numbers NP_001095202, NP_001095201 and NP_001075171 respectively) was calculated. Protein sequences of chicken collagens IV, V and X (NCBI Accession Numbers XP_422615, NP_990121 and AAA48736 respectively) were used. Protein sequence of Elastin was downloaded from the ElastoDB and used for calculation of amino acid composition.
(TIFF)

Figure S3 **Protein sequence of the CREMP-2Rep construct.** Non-CREMP amino acid residues that are contributed by the pTrc His A vector are underlined. The hexa-histidine tag is highlighted in pink. The TEV protease site is shown in grey, and the GG dipeptide, introduced for efficient TEV protease cleavage, is depicted in black.
(TIFF)

Figure S4 **Fluorescence spectroscopy of CREMP-2Rep.** CREMP (5 µM in 50 mM phosphate buffer, pH 7.5 containing 1 mM EDTA) was incubated with 1 mM THP and fluorescence emission spectra, exciting at 280 nm, were recorded.
(TIFF)

Figure S5 **Oxidation of reduced CREMP-2Rep by chicken QSOX.** Reduced CREMP-2Rep was prepared by incubating 100 µM protein with 40 mM THP for 2h at 25°C in 50 mM phosphate buffer pH 7.5 containing 1 mM EDTA. The reduced protein was applied to a PD10 gel filtration column and eluted with the same buffer. In panel A, reduced CREMP (19 µM protein, 304 µM thiols) was incubated with, or without, 50 nM avian QSOX (solid and open squares, respectively). Aliquots of the reaction mixture were removed at the times indicated for discontinuous sampling with DTNB [27]. Panel B shows the increase in fluorescence excited at 280 nm when 10 nM QSOX is added to 5 µM reduced CREMP in phosphate buffer (pH 7.5, 25°C). Reduced and oxidized spectra (red and green curves, respectively) were collected 12 min apart.
(TIFF)

Figure S6 **Cladogram comparing a and b modules of chicken CREMP.** All available chicken CREMP repeats in the (a-b)_n_ format were aligned using ClustalW and a phylogenetic tree was constructed using FigTree (**a** and **b** modules are depicted in red and blue).
(TIFF)

Figure S7 **Zebra finch CREMP.** Sequences of CREMP repeats derived from a short central region of the contig NW_002229169 are shown below. Cysteine residues are highlighted and the protein sequence is arranged to depict three complete CREMP repeats.
(TIFF)

Table S1 **Amino acid composition of chicken ESM taken from the literature.**
(DOC)

Table S2 **List of Primers used in this study.**
(DOC)

Table S3 **Peptides Isolated from alkylated avian ESM.** The preparation and sequencing of chicken ESM peptides was performed as described in Materials and Methods.
(DOC)
