# Supplementary figures and images for: A Novel Disulfide-Rich Protein Motif from Avian Eggshell Membranes
Source: PLoS One. 2011 Mar 30;6(3):e18187. doi: 10.1371/journal.pone.0018187 (PMC3068167; doi:10.1371/journal.pone.0018187)

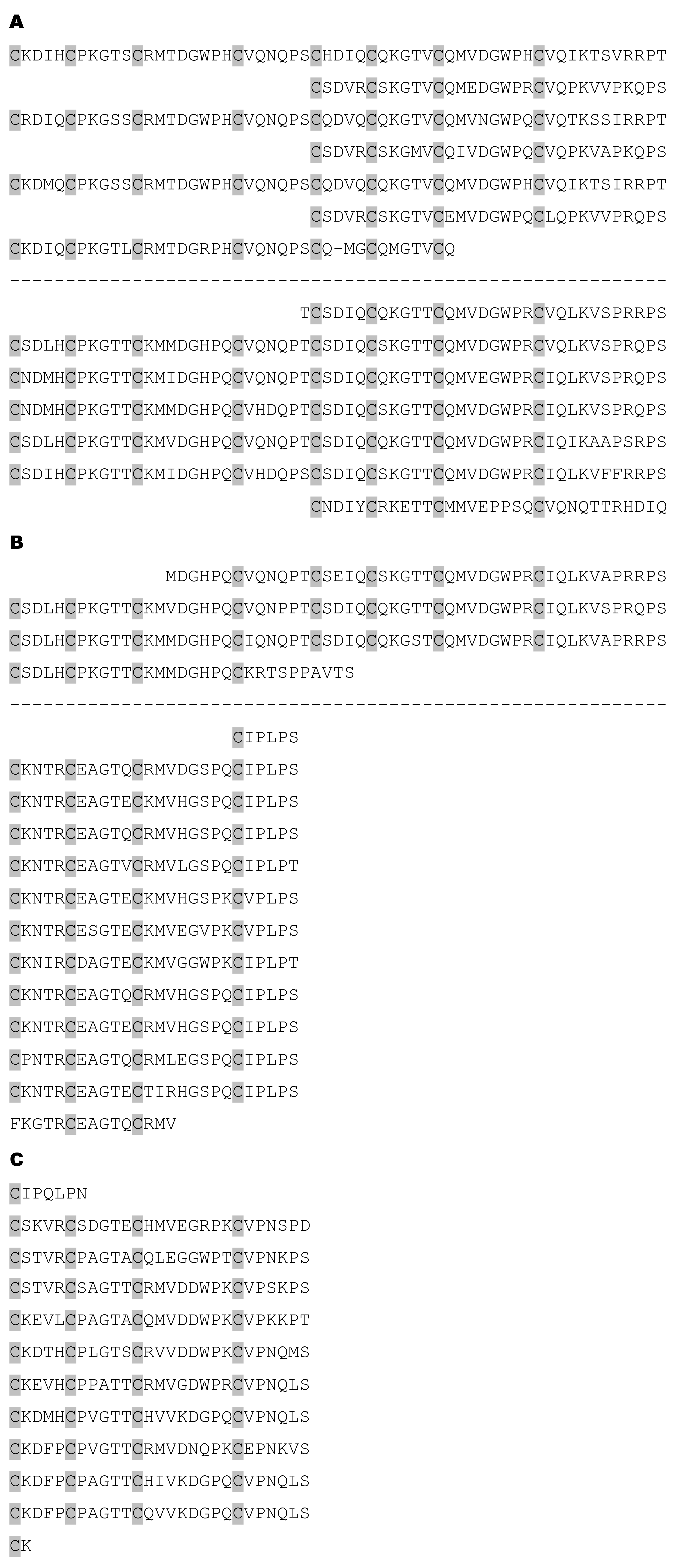

Supplement: Figure S1 — CREMP sequences derived from different Gallus gallus contigs. The sequences shown in A, B and C are derived from the contigs NW_001473877, NW_001479668 and NW_1475627 respectively. The dashed lines separating sequences in A and B represent a stretch of unassigned nucleotides in the contig. The sequence shown in the second part of A is deposited in the RefSeq database under the accession number XP_001236415. (TIFF) [file pone.0018187.s001.tiff]

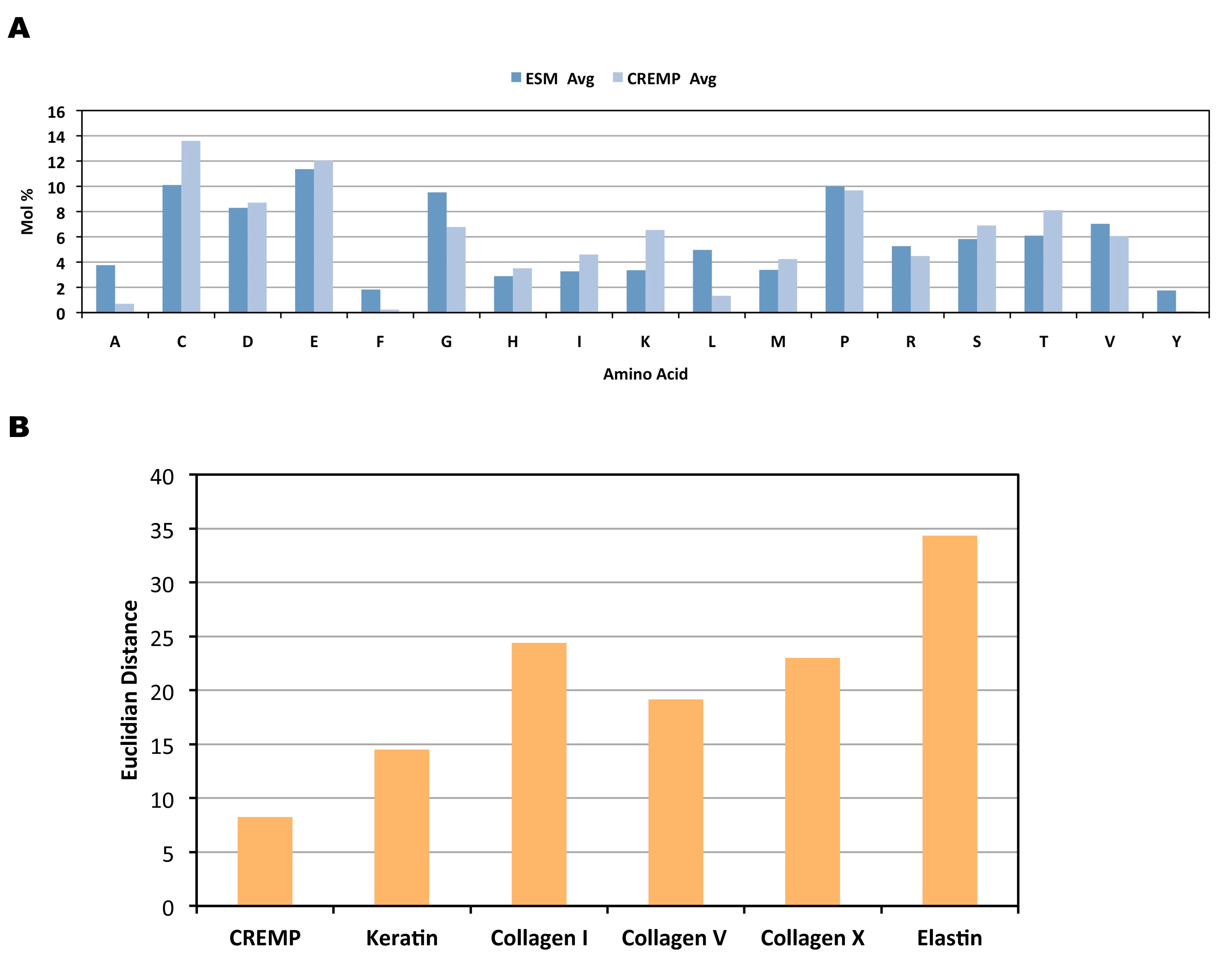

Supplement: Figure S2 — Comparison of the amino acid composition between chicken ESM and CREMP repeats and of CREMP to other structural proteins. Panel A: For chicken ESMs, an average of the literature values for amino acid composition in Table S1 was used. For CREMP, all available repeats were summed using the ProtParam tool (http://expasy.org/tools/protparam.html) to calculate the amino acid composition. To compare data obtained by acid hydrolysis with compositions deduced from gene sequences the content of ASP and ASN and GLU and GLN are aggregated. Where available, the amounts of Hyl and Hyp were added to Lys and Pro, respectively. Trp was not included because amino acid analyses for this amino acid were unavailable. Panel B: Euclidian distances between average amino acid composition of ESMs and other proteins were calculated as described previously [51]. The values for chicken ESM and for CREMP were as in panel A. For keratin, an average amino acid composition of chicken feather keratins 1, 3 and 4 (RefSeq Accession Numbers NP_001095202, NP_001095201 and NP_001075171 respectively) was calculated. Protein sequences of chicken collagens IV, V and X (NCBI Accession Numbers XP_422615, NP_990121 and AAA48736 respectively) were used. Protein sequence of Elastin was downloaded from the ElastoDB and used for calculation of amino acid composition. (TIFF) [file pone.0018187.s002.tiff]

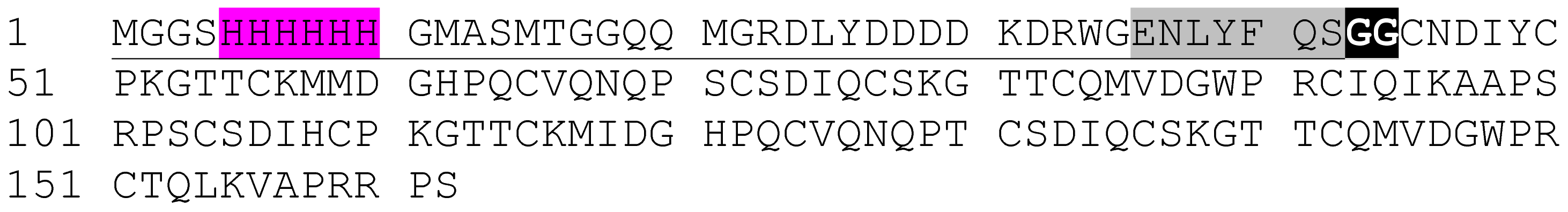

Supplement: Figure S3 — Protein sequence of the CREMP-2Rep construct. Non-CREMP amino acid residues that are contributed by the pTrc His A vector are underlined. The hexa-histidine tag is highlighted in pink. The TEV protease site is shown in grey, and the GG dipeptide, introduced for efficient TEV protease cleavage, is depicted in black. (TIFF) [file pone.0018187.s003.tiff]

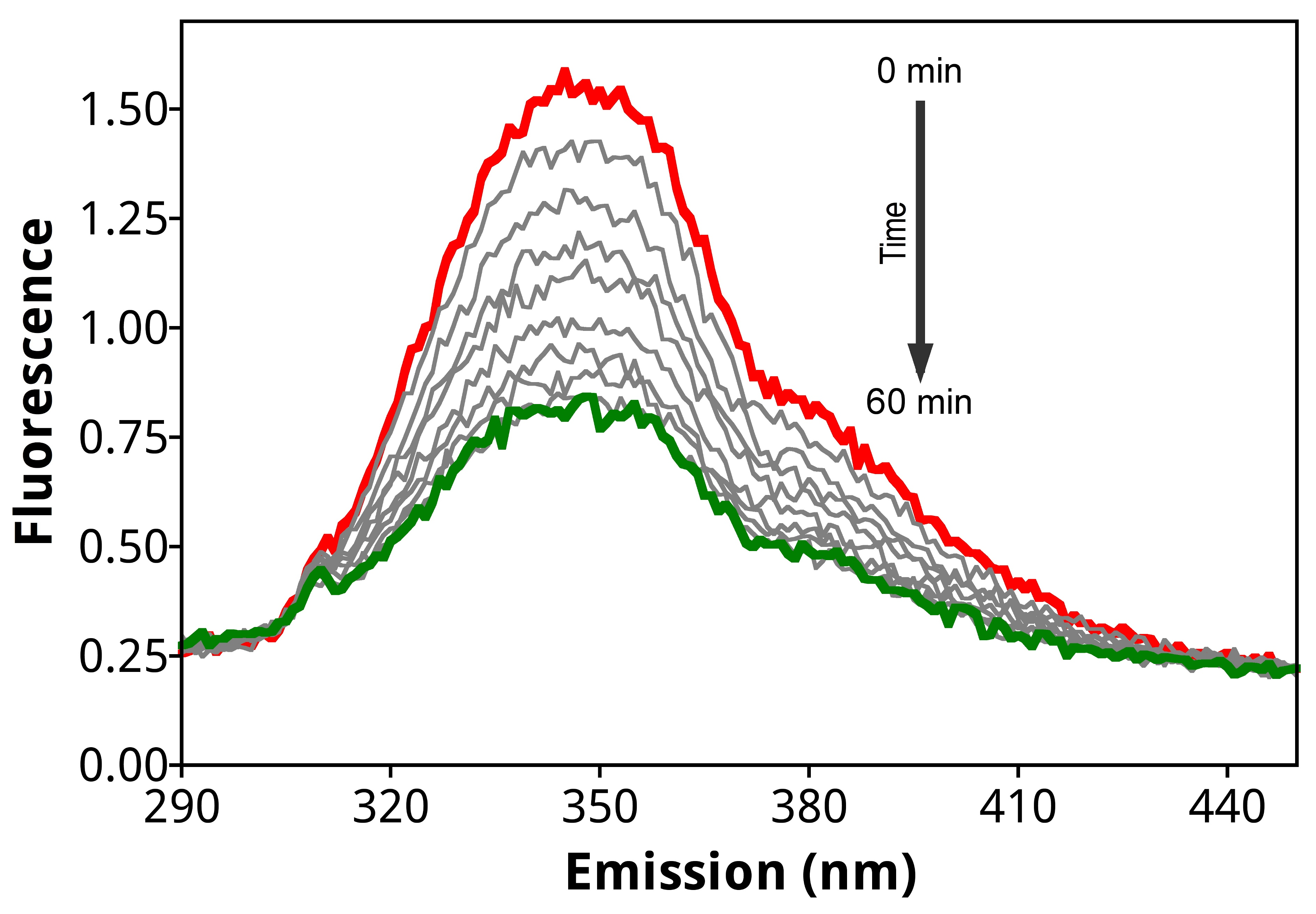

Supplement: Figure S4 — Fluorescence spectroscopy of CREMP-2Rep. CREMP (5 µM in 50 mM phosphate buffer, pH 7.5 containing 1 mM EDTA) was incubated with 1 mM THP and fluorescence emission spectra, exciting at 280 nm, were recorded. (TIFF) [file pone.0018187.s004.tiff]

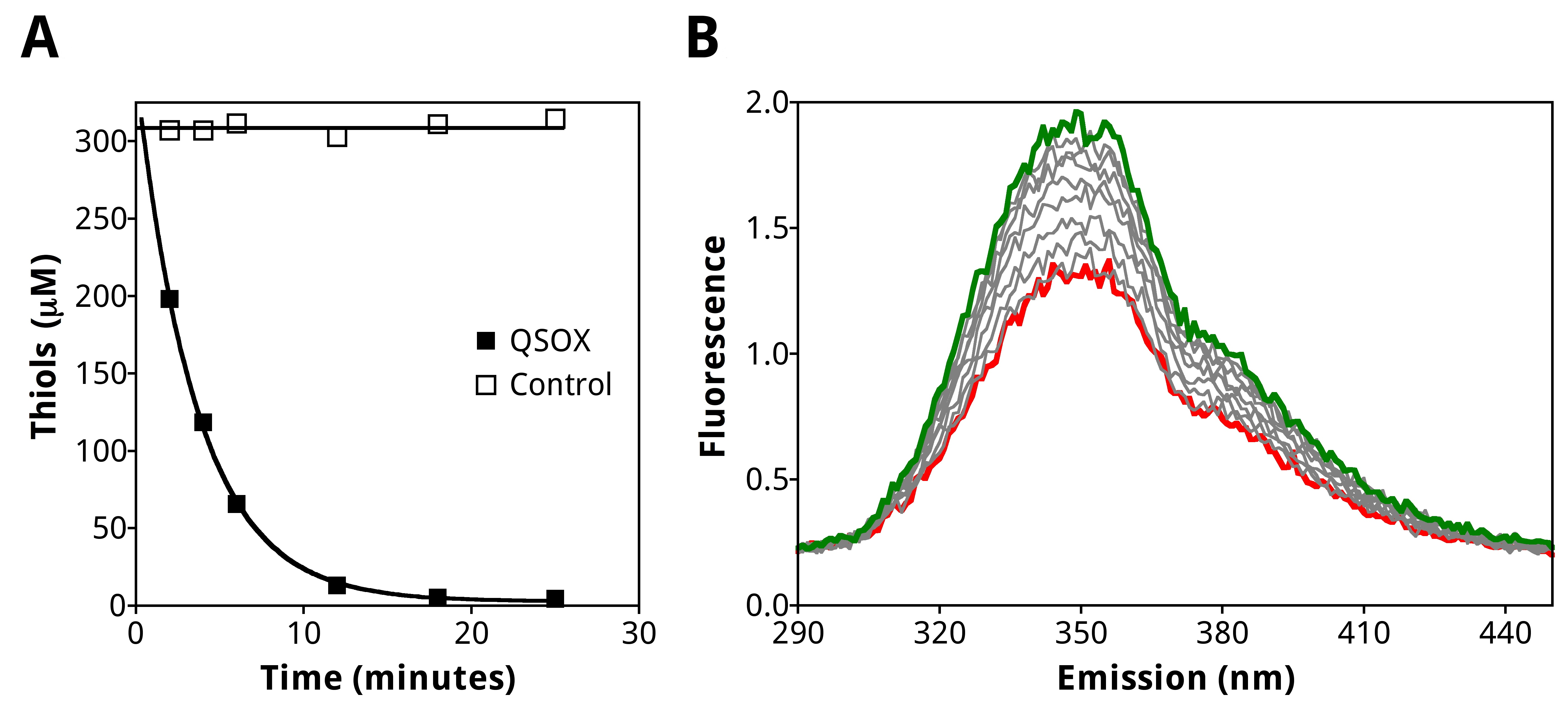

Supplement: Figure S5 — Oxidation of reduced CREMP-2Rep by chicken QSOX. Reduced CREMP-2Rep was prepared by incubating 100 µM protein with 40 mM THP for 2h at 25°C in 50 mM phosphate buffer pH 7.5 containing 1 mM EDTA. The reduced protein was applied to a PD10 gel filtration column and eluted with the same buffer. In panel A, reduced CREMP (19 µM protein, 304 µM thiols) was incubated with, or without, 50 nM avian QSOX (solid and open squares, respectively). Aliquots of the reaction mixture were removed at the times indicated for discontinuous sampling with DTNB [27]. Panel B shows the increase in fluorescence excited at 280 nm when 10 nM QSOX is added to 5 µM reduced CREMP in phosphate buffer (pH 7.5, 25°C). Reduced and oxidized spectra (red and green curves, respectively) were collected 12 min apart. (TIFF) [file pone.0018187.s005.tiff]

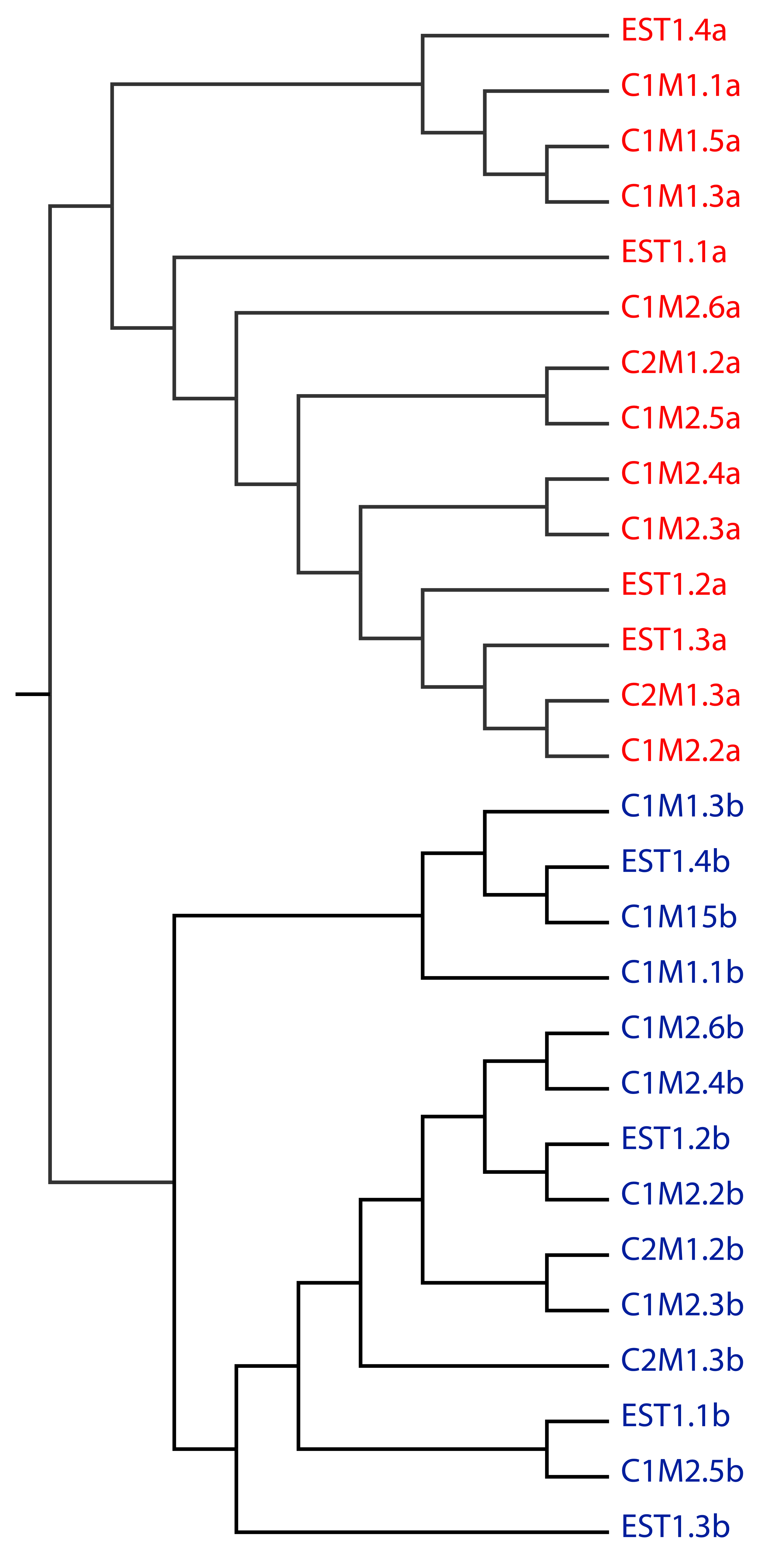

Supplement: Figure S6 — Cladogram comparing a and b modules of chicken CREMP. All available chicken CREMP repeats in the (a-b)n format were aligned using ClustalW and a phylogenetic tree was constructed using FigTree (a and b modules are depicted in red and blue). (TIFF) [file pone.0018187.s006.tiff]

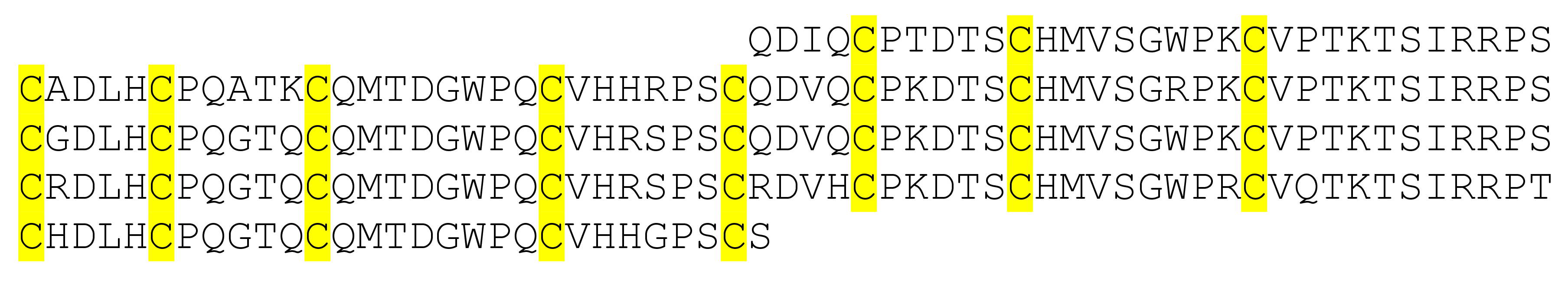

Supplement: Figure S7 — Zebra finch CREMP. Sequences of CREMP repeats derived from a short central region of the contig NW_002229169 are shown below. Cysteine residues are highlighted and the protein sequence is arranged to depict three complete CREMP repeats. (TIFF) [file pone.0018187.s007.tiff]
