# Supplementary material for: A Novel Disulfide-Rich Protein Motif from Avian Eggshell Membranes
Source: PLoS One. 2011 Mar 30;6(3):e18187. doi: 10.1371/journal.pone.0018187 (PMC3068167; doi:10.1371/journal.pone.0018187)
Supplement: Table S1 — Amino acid composition of chicken ESM taken from the literature. (DOC) [file pone.0018187.s008.doc]

**Table S1. Amino acid composition of chicken ESM taken from the literature.**

| **Residue** | **Ref. [1]** | **Ref. [2]** | **Ref. [3]** | **Ref. [4]** | **Ref. [5]** | **Ref. [6]** | **Average±SD** |
| --- | --- | --- | --- | --- | --- | --- | --- |
| **Alanine** | 4 | 4 | 3.9 | 4.1 | 2.3 | 4.2 | 3.7±0.7 |
| **Arginine** | 5.5 | 5.1 | 4.8 | 5.2 | 5.9 | 5.1 | 5.3±0.4 |
| **Aspartic acid** | 7.5 | 8.1 | 6.1 | 8.2 | 11.7 | 8.2 | 8.3±1.8 |
| **Cysteine** | 10.3 | 10 | 8.8 | 10.4 | 10.9 | 10.2 | 10.1±0.7 |
| **Glutamic acid** | 9.7 | 11 | 10.2 | 10.5 | 16 | 10.8 | 11.4±2.3 |
| **Glycine** | 9.7 | 10.3 | 11.9 | 10.4 | 4 | 10.9 | 9.5±2.8 |
| **Histidine** | 3.3 | 3.1 | 4.1 | 2.9 | 1.3 | 2.6 | 2.9±0.9 |
| **Hydroxylysine** | 0.1 | nd | 3.3 | nd | nd | 0.2 | 1.2±1.8 |
| **Hydroxyproline** | 0.9 | nd | 0.6 | nd | nd | 1.3 | 0.9±0.3 |
| **Isoleucine** | 3.6 | 3.2 | 3 | 3.3 | 3.8 | 2.6 | 3.2±0.4 |
| **Leucine** | 4.8 | 4.7 | 4.8 | 5 | 6.1 | 4.4 | 5.0±0.6 |
| **Lysine** | 3.4 | 3.1 | 3.9 | 3 | 3.9 | 2.9 | 3.4±0.4 |
| **Methionine** | 3 | 3.4 | 2 | 3.6 | 4.7 | 3.6 | 3.4±0.9 |
| **Phenylalanine** | 1.5 | 1.4 | 1.9 | 1.7 | 3.6 | 0.9 | 1.8±0.9 |
| **Proline** | 11.9 | 10.5 | 8.9 | 9.8 | 8.4 | 10.5 | 10.0±1.3 |
| **Serine** | 6 | 6.8 | 6.4 | 6.7 | 1.7 | 7.4 | 5.8±2.1 |
| **Threonine** | 5.7 | 6.2 | 6.1 | 6.2 | 6.2 | 6.2 | 6.1±0.2 |
| **Tyrosine** | 1.2 | 1.3 | 1.5 | 1.5 | 3.9 | 1.1 | 1.7±1.1 |
| **Valine** | 7.9 | 7.9 | 7.8 | 7.5 | 5.6 | 5.6 | 7.0±1.1 |

**References**

1. Leach RM, Jr., Rucker RB, Van Dyke GP (1981) Egg shell membrane protein: a nonelastin desmosine/isodesmosine-containing protein. Arch Biochem Biophys 207: 353-359.

2. Baumgartner S, Brown DJ, Salevsky E, Jr., Leach RM, Jr. (1978) Copper deficiency in the laying hen. J Nutr 108: 804-811.

3. Blake JP, Kling LJ, Halteman WA (1985) The Relationship of the Amino-Acid Composition of a Portion of the Outer Eggshell Membrane to Eggshell Quality. Poultry Science 64: 176-182.

4. Salevsky E, Leach RM (1980) Studies on the Organic-Components of Shell Gland Fluid and the Hens Eggshell. Poultry Science 59: 438-443.

5. Wedral EM, Vadehra DV, Baker RC (1974) Chemical composition of the cuticle, and the inner and outer shell membranes from eggs of Gallus gallus. Comp Biochem Physiol B 47: 631-640.

6. Crombie G, Snider R, Faris B, Franzblau C (1981) Lysine-Derived Cross-Links in the Eggshell Membrane. Biochimica et Biophysica Acta 640: 365-367.
