# Supplementary material for: A Novel Disulfide-Rich Protein Motif from Avian Eggshell Membranes
Source: PLoS One. 2011 Mar 30;6(3):e18187. doi: 10.1371/journal.pone.0018187 (PMC3068167; doi:10.1371/journal.pone.0018187)
Supplement: Table S2 — List of Primers used in this study. (DOC) [file pone.0018187.s009.doc]

**Table S2 List of primers used in this study.**

| **Primer Name** | **DNA Sequence** |
| --- | --- |
| CREMP-2Rep-pTrc-F-*Bam*HI | TTAGCGGGATCCTGCAATGACATCTACTGTCCC |
| CREMP-2Rep-pTrc-R-*Eco*RI | GAATTCTCAAGAAGGTCGCCTTGGGGC |
| CREMP-TEV-1F | GATAAGGATCGATGGGGAGAGAACCTGTCCTGCAATGACATCTAC |
| CREMP-TEV-1R | GTAGATGTCATTGCAGGACAGGTTCTCTCCCCATCGATCCTTATC |
| CREMP-TEV-2F | GATGGGGAGAGAACCTGTACTTCCAGTCCTGCAATGACATCTAC |
| CREMP-TEV-2R | GTAGATGTCATTGCAGGACTGGAAGTACAGGTTCTCTCCCCATC |
| CREMP-TEV-2G-F | CTGTACTTCCAGTCCGGTGGTTGCAATGACATCTAC |
| CREMP-TEV-2G-R | GTAGATGTCATTGCAACCACCGGACTGGAAGTACAG |
