# Supplementary material for: A Novel Disulfide-Rich Protein Motif from Avian Eggshell Membranes
Source: PLoS One. 2011 Mar 30;6(3):e18187. doi: 10.1371/journal.pone.0018187 (PMC3068167; doi:10.1371/journal.pone.0018187)
Supplement: Table S3 — Peptides Isolated from alkylated avian ESM. The preparation and sequencing of chicken ESM peptides was performed as described in Materials and Methods. (DOC) [file pone.0018187.s010.doc]

**Table S3 Peptides isolated from alkylated avian ESM.** The preparation and sequencing of chicken ESM peptides was performed as described in Materials and Methods.

| **Protein Name** | **Accession** | **Peptide Sequences** |
| --- | --- | --- |
| **CREMP**  **Similar to spore coat protein SP75** | XP_001236415 | - MVDGWPR - CPAGTACQLEGGWPTCVPNKPSCSTVR - MVDGWPRC - CPAGTACQMVDDWPK - RPSCSDLHCPK - IPSCQDLQCPSGTACR - QPSCSDLHCPK - VDGWPR - CVPNSPDCSTVR - GTTCQMVDGWPR - GTTCKMMDGHPQCVQNQPTCSDIQCSKGTTCQMVDGWPR - VVDDWPK |
| **Ovocalyxin-36** | NP_001026032  CAI91279 | - LPRLNAVAADGIPLPDVF - LNAVAADGIPLPDVF - VECVGNPILL |
| **Collagen X (alpha 1)** | P08125  AAA48736 | - AYPGATVPIKF - LDQASGSAVIDLMENDQVW - LQLPNSESNGLY - LDQASGSAVIDL - AYPGATVPIK |
| **Lysyl Oxidase** | XP_423667 | - ELGFGSAK - FGAGVSCSETAPDLVL - VWHDCHR - QVVINPNYEVAESDY - NLVSASVVCR - LGQGMGPIHL - LEDRPMF - TVCEKAVPVTE - YNCHTGGSRD |
| **Lysozyme** | P00698 | - CNIPCSALL - VCAAKF |
| **Protein Phosphatase 1J** | XP_418000 | - SQAEDNGEVPNDALPR - ARVMATIGVTR |
| **Similar to Transmembrane Protein 151B-like** | XP_426130 | - NHDHRHL - AVLDIRSPR |
| **Bromodomain containing protein 8** | NP_001006148  CAG32596 | - LQPVTDDIAPGYHSIVQRPMDL - ETTETPK |
| **Hypothetical Protein** | XP_001236449 | - SRGEGAGNSAGAVGKGAGK - SPRAAQVL |
| **similar to calcium channel, voltage-dependent, alpha 1H subunit** | XP_414830 | - TDSVDGHPPDPK - GQVLAATMSDGESREPRPAGGEVR |
| **similar to Trpv2 protein**  **transient receptor potential cation channel, subfamily V, member 2** | XP_415848 | - KEGVCFYFGELPLSLAACTNQFEVVEYLLNNPHQK - VTLMGDAPSLSQNK |
| **similar to polycystic kidney and hepatic disease 1** | XP_420050 | - CGESQAQQQQGDTGPSQSHVVGLIASPK - NVTCIAIQCTVPPGNGTRALR |
| **protocadherin-1** | NP_001039292  AAT74929 | - VQVVDVNDNAPVFSQSFTEVAFPENNEPNDL - ITIVDMNDNAPK |
| **PREDICTED: similar to splicing factor 3b, subunit 1** | XP_421912 | - RTAVSPPLPPPPSER - TPGKTPIGTPAMNMATPTPGHIMSMTPEQL |
| **PREDICTED: similar to myeloid/lymphoid or mixed-lineage leukemia 3** | XP_418542 | - VIGNGPGQESAVGCVTDASTSHKK - NGVGTVVIPGVTTMDISSNKDEEENSMHNTVVL |
| **PREDICTED: similar to anchor protein** | XP_414354 | - NTSVLSSVQSSSTPSGPQTTANPSGMHTSTPSAMCPSTPHSSTSHSISEL - GSDTCLPCDCYPVGSSSRSCNKETGR |
| **PREDICTED: similar to neuron navigator 2** | XP_420909  XP_427719 | - EMKLTDIR - GIGGLNSSNSSQSVSGPATTHSTGSNTVSVQLPQPQQQYSHPNTATVAPF |
| **dnaJ homolog subfamily C member 7** | NP_001026673  CAH65159 | - RMDSTNADAL - LGRYPEAQSVASDIL |
| **PREDICTED: similar to gravin** | XP_419673 | - EDDTQTMETSPSDSSTKDGVAAEKDAHTDK - EEQEKEGATLTTETSEK |
| **Similar to mediator of RNA polymerase II transcription subunit 15** | XP_415235 | - SVPADYPDQSPLWIK - GPMGQQMSLPGQQQPPGSTGMAPHGMPGVSTATQQTQL |
| **Hypothetical protein similar to FAM21A** | XP_001234527  CAH65442 | - TSEEGSVDSDRGSVLDSEEK - NSPISFLEEEEDFLFTSQKTGKK |
| **PREDICTED: similar to KIAA1525 protein**  **BTB/POB domain containing protein** | XP_421333 | - TVDTELSQTVTEVGPGPPQHISCIQSR - GAEIIMDINTAGIDMPMFSAL |
| **PREDICTED: similar to mtprd** | XP_416727 | - LPAVSSSEDQGPTTASF - TRSFIRVLSEMEEVDPK |
| **PREDICTED: hypothetical protein** | XP_416578 | - CTVEAPGDTSGDPDKEVK - QDVVVTWR |
| **PREDICTED: similar to titin isoform N2-A** | XP_001234116 BAF64427 | - TLENPVSSVSGEIDVNVIARPSAPK - VGETARFEIEISEPDVHPIW |
| **zinc finger protein ZFP** | NP_990541 | - QNSEAASSSGLENGTNESLAEPSVSQSDSENK - VTSQSTPSCELQNSEAASSSGL |
| **PREDICTED: similar to UBE3B ubiquitin protein ligase** | XP_423951  XP_001235595  AAG53076 | - SIRCVEVSDDQDTGDTL - GVDEAGIDQDGVFKEF |
| **PREDICTED: similar to QSER1 protein** | XP_419636 | - SEESAASENDFNMSGDDGTVAGNQSK - ASAVPSSGFPPASAAK |
| **PREDICTED: similar to C219-reactive peptide (FLJ39207)** | XP_419399 | - GAADAGESAKGDTAPDHVPGVPPSLPPDPGAVPPGGGASEGALSGAVR - SAAQNTNAENSTQQGTAHTDNEDSDR |
